# *Anoectochilus roxburghii* Extract Extends the Lifespan of *Caenorhabditis elegans* through Activating the *daf-16*/FoxO Pathway

**DOI:** 10.3390/antiox13080945

**Published:** 2024-08-02

**Authors:** Peng Xu, Jianfeng Wang, Junyi Wang, Xiaoxiao Hu, Wei Wang, Shengmin Lu, Yingkun Sheng

**Affiliations:** 1 Xingzhi College, Zhejiang Normal University, Jinhua 321100, China; 2022111026061@stu.hznu.edu.cn (P.X.); jeffwong34@zjnu.edu.cn (J.W.); xxhu@zjnu.cn (X.H.); 2 School of Basic Medical Science, Hangzhou Normal University, Hangzhou 311121, China; 3 Life Sciences, Zhejiang Normal University, Jinhua 321017, China; junyiwang@zjnu.edu.cn; 4 Taizhou Research Institute, Southern University of Science and Technology, Taizhou 317700, China; awei@sustechtz.com; 5 State Key Laboratory for Managing Biotic and Chemical Threats to the Quality and Safety of Agro-Products, Institute of Food Science, Zhejiang Academy of Agricultural Sciences, Hangzhou 310021, China

**Keywords:** *Anoectochilus roxburghii*, *Caenorhabditis elegans*, *daf-16*/FoxO pathway, anti-aging, lifespan, stress resilience

## Abstract

As a significant global issue, aging is prompting people’s interest in the potential anti-aging properties of *Anoectochilus roxburghii* (*A. roxburghii*), a plant traditionally utilized in various Asian countries for its purported benefits in treating diabetes and combating aging. However, the specific anti-aging components and mechanisms of *A. roxburghii* remain unclear. This study aims to investigate the anti-aging effects and mechanisms of *A. roxburghii* extract E (ARE). *Caenorhabditis elegans* (*C. elegans*) were exposed to media containing different concentrations of ARE whose superior in vitro radical scavenging capacity was thus identified. Lifespan assays, stress resistance tests, and RT-qPCR analyses were conducted to evaluate anti-aging efficacy, reactive oxygen species (ROS) levels, antioxidant enzyme activity, and *daf-16*, *sod-3*, and *gst-4* levels. Additionally, transcriptomic and metabolomic analyses were performed to elucidate the potential anti-aging mechanisms of ARE. Fluorescence protein assays and gene knockout experiments were employed to validate the impacts of ARE on anti-aging mechanisms. Our results revealed that ARE not only prolonged the lifespan of *C. elegans* but also mitigated ROS and lipofuscin accumulation, and boosted resistance to UV and heat stress. Furthermore, ARE modulated the expression of pivotal anti-aging genes including *daf-16*, *sod-3*, and *gst-4*, facilitating the nuclear translocation of DAF-16. Significantly, ARE failed to extend the lifespan of *daf-16*-deficient *C. elegans* (CF1038), indicating its dependency on the *daf-16*/FoxO signaling pathway. These results underscored the effectiveness of ARE as a natural agent for enhancing longevity and stress resilience to *C. elegans*, potentially to human.

## 1. Introduction

Aging is a complex biological process characterized by the gradual decline in the physiological functions of tissues and organs, as well as a loss of repair capabilities, which increases the risk to various diseases [1,2,3]. To delay aging, several strategies have been proposed, including calorie restriction, regular exercise, and dietary supplementation [4,5]. Recently, there has been a marked increase in interest in identifying natural compounds that can delay aging and extend lifespan. Among the range of natural products, medicinal plants have shown considerable promise due to their bioactive compounds which offer potential health benefits [6,7,8,9].

*Caenorhabditis elegans* (*C. elegans*) has emerged as a valuable model organism in aging research due to its short lifespan, well-defined genetics, and highly conserved aging pathways similar to those in mammals [10,11]. The *daf-16*/FoxO signaling pathway in *C. elegans* plays a pivotal role in regulating lifespan and stress resistance, making it an exemplary system for exploring the molecular mechanisms of potential anti-aging compounds [12,13,14]. Previous research has demonstrated that natural compounds can modulate the *daf-16*/FoxO pathway, effectively enhancing the lifespan and stress resilience of *C. elegans* [15,16,17].

*Anoectochilus roxburghii (A. roxburghii)*, a rare orchid species, is widely found in tropical and subtropical regions of Asia, particularly in mountainous areas of southern China [18]. As a traditional medicinal herb, it is highly valued in Asian countries, especially in China and Japan, for its numerous therapeutic properties. Notably, *A. roxburghii* has significant anti-inflammatory effects and is traditionally used to treat inflammation, detoxify the body, and alleviate throat infections and bronchitis [19]. Additionally, it is believed to strengthen the immune system, enhance the body’s ability to combat diseases, and protect the liver, aiding in the treatment of hepatitis and other liver conditions [20,21,22].

Recent advances in science and technology have led to a deeper understanding of the bioactive components of *A. roxburghii* and their potential health benefits. Research has indicated that it is rich in antioxidants, such as flavonoids and polysaccharides, which can scavenge free radicals and reduce oxidative stress, thereby potentially slowing the aging process [23]. Additionally, *A. roxburghii* extract has also been found to lower blood sugar levels through various mechanisms, including enhancing insulin sensitivity and improving glucose metabolism [24]. However, the specific effects of *A. roxburghii* extract on the lifespan, stress resistance, and underlying molecular mechanisms in *C. elegans* have not been extensively explored.

This study employed lifespan assays, stress resistance tests, transcriptomics, and metabolomics to examine the influence of *A. roxburghii* extract on the lifespan, stress resistance, gene expression, and to elucidate the potential mechanisms underpinning the observed phenotypic changes in *C. elegans*. The results would provide a theoretical foundation for the development of anti-aging health products based on *A. roxburghii*.

## 2. Materials and Methods

### 2.1. Reagents and Materials

*E. coli* OP50 and *C. elegans* (N2, TJ356, CF1553, CL2166, CF1038) were acquired from the Caenorhabditis Genetics Center (CGC, Minneapolis, MN, USA). *A. roxburghii* was provided from the Jinhua Academy of Agricultural Sciences (Jinhua, Zhejiang, China). Plant specimens were preserved at the Southern Agriculture and Forestry Resources Laboratory of Xingzhi College, Zhejiang Normal University. The following reagents were utilized in the experiments: agar, KH_2_PO_4_, Na_2_HPO_4_, NaH_2_PO_4_, NaCl, NaClO, CaCl_2_, NaOH, MgSO_4_, cholesterol, yeast extract, tryptone, and peptone were purchased from Sangon Biotech (Shanghai, China). TB Green^®^ Premix Ex Taq™ and PrimeScript™ RT reagent Kit with gDNA Eraser were supplied by Takara (Beijing, China). Commercial kits for measuring SOD, MDA, CAT, and GSH-px were provided by the Nanjing Institute of Biological Engineering (Nanjing, China). Reactive Oxygen Species Assay Kit and Detergent Compatible Bradford Protein Assay Kit were obtained from Beyotime (Shanghai, China). A BCA assay kit was purshased from Nanjing Institute of Biological Engineering (Nanjing, China).

### 2.2. Preparation and Identification of AREs

The *A. roxburghii* were divided into five categories based on different polarities: A, B, C, D, and E. The detailed methods for extraction and screening are provided in Appendix A. The *A. roxburghii* fraction E, named ARE, had exhibited superior antioxidant properties compared to other fractions during in vitro antioxidant experiments. Subsequently, the LC-MS was used to identify the components of ARE and the condition as follows.

After freeze-drying, 8 mg ARE were weighed into a 1.5 mL EP tube, two small steel balls were added, and 400 μL methanol-water (*v*:*v* = 4:1, including mixed internal standard, 4 μg/mL) was added. After pre-cooling for 2 min in the −40 °C refrigerator, it was put it into the grinder for grinding (60 Hz, 2 min). It then underwent ultrasonic extraction in an ice water bath for 10 min and was left overnight at −40 °C. Then the sample was centrifuged for 10 min (12,000 rpm, 4 °C), extracted 150 μL supernatant with a syringe, filtered with 0.22 μm organic phase pinhole filter, transfered to LC sample vial, and stored at −80 °C until LC-MS analysis.

Metabolites were separated and detected using a liquid chromatography-mass spectrometry system composed of a Waters (Milford, MA, USA) ACQUITY UPLC I-Class plus and Thermo (Waltham, MA, USA) QE high-resolution mass spectrometer. The chromatographic separation was performed on an ACQUITY UPLC HSS T3 column (100 mm × 2.1 mm, 1.8 μm) with the following gradient program: column temperature at 45 °C; mobile phase A (water containing 0.1% formic acid) and mobile phase B (acetonitrile); flow rate at 0.35 mL/min with a linear gradient setting as follows: 5% B (0–2 min), 5–30% B (2–4 min), 50% B (4–8 min), 50–80% B (8–10 min), 80–100% B (10–14 min), 100–100% B (14–15 min), 100–5% B (15–15.1 min), 5% B (15.1–16 min).

The mass spectra were acquired ranging from 70 to 1050 m/z with a primary resolution of 70,000 and a secondary resolution of 17,500. The sheath gas flow rate was 35, and the auxiliary gas flow rate was 8. The spray voltages were set at 3.8 kV (positive ion) and 3 kV (negative ion). The capillary temperature was maintained at 320 °C, and the auxiliary gas heater temperature was at 350 °C. Progenesis QI v3.0 software (Nonlinear Dynamics, Newcastle, UK) was used for baseline filtering, peak recognition, integration, retention time correction, peak alignment, and normalization of the original data.

### 2.3. Culture and Treatment of C. elegans

The *C. elegans* were cultured at 20 °C in nematode growth media (NGM) with *E. coli* OP50 serving as the food source and treated with sodium hypochlorite to synchronize the populations. The protocols for cultivation and maintenance followed those outlined in the WormBook [25]. The dosed plates were prepared by adding 10 μg/mL and 100 μg/mL ARE into the NGM and then coated with OP50 on the surface and incubated at 37 °C for 12 h. 

Subsequently, synchronized *C. elegans* (L1 stage) were cultured at 20 °C for 3 days in NGM with OP50, water, 10 μg/mL, and 100 μg/mL ARE to evaluate the toxicity of ARE. The body length, locomotion, and pumping rates of the *C. elegans* were measured using an Mshot ML31 biomicroscope with an MShot Image Analysis System (Guangzhou Micro-shot Technology Co., Guangzhou, China). Statistical analysis was performed using a Student’s *t*-test. The data are presented as mean ± SD.

Furthermore, to evaluate the preference of *C. elegans* for OP50 mixed with ARE, 50 μL of OP50 alone and 50 μL of OP50 mixed with 100 μg/mL ARE were seeded on opposite sides of an NGM plate. Then, 200 worms were placed in the center of the plate. After 1 h, the number of worms on each bacterial lawn was counted. This experiment was repeated three times.

### 2.4. Lifespan Analysis

Synchronized L4 stage *C. elegans* were picked and placed on standard NGM plates containing different concentrations of ARE, with ample OP50 provided as food. The NGM plates also contained 100 μM 5-fluoro-2-deoxyuridine (FUDR). The *C. elegans* were observed every 24 h until all had died.

After culturing synchronized L4 stage *C. elegans* for 2 days, they were subjected to UV irradiation and heat treatment at 37 °C to induce stress responses, which differ slightly from conventional lifespan assays. The UV treatment group was monitored every 24 h, whereas the heat treatment group was observed every hour until all had died. The data were statistically analyzed using the log-rank test.

### 2.5. Lipofuscin Level Assay

L4 stage larvae were incubated with 0 μg/mL, 10 μg/mL, or 100 μg/mL ARE. In 7-day-old worms, the autofluorescence of intestinal lipofuscin was measured through the use of a fluorescence microscopy (Guangzhou Micro-shot Technology Co., Guangzhou, China). The fluorescence intensity of 20 worms was quantified using the Image J software (version 15.4f) to determine lipofuscin levels.

### 2.6. Stress Tolerance Tests

The L4 stage *C. elegans* were treated with water, 10 μg/mL or 100 μg/mL ARE for 48 h, and then randomly divided into the control and treated groups with 200 worms per group. Afterward, the *C. elegans* were exposed to 100 mj of energy under a UV for 5 min to evaluate the UV protection by ARE.

In addition, the *C. elegans* were incubated at 37 °C, and their death number was recorded hourly to evaluate the resistance to heat stress of ARE.

In each experiment, death was determined by a lack of response to touch with a platinum wire. Unexpected deaths, such as those from loss or wall climbing, were excluded. Statistical analysis of the data was performed using the log-rank test.

### 2.7. Determination of ROS and Antioxidant Enzymes

Synchronized L4 stage *C. elegans* were treated in the NGM containing water, 10 μg/mL and 100 μg/mL ARE for 48 h, respectively. Subsequently, M9 buffer was used to wash *C. elegans* 3 times. Then, the active oxygen probe (DCFH-DA) was added at a ratio of 1:1000 and incubated at 20 °C for 25 min. After incubation, *C. elegans* were washed three times with M9 buffer and then placed on 2% agarose pads for photographing. ROS levels were measured using a microplate reader (Spark03030923, Tecan, Mannedorf, Switzerland) at an excitation wavelength of 488 nm and an emission wavelength of 525 nm. 

Synchronized L4 stage *C. elegans* were transferred onto NGM containing 0 μg/mL, 10 μg/mL, and 100 μg/mL ARE, respectively. A total of 500 *C. elegans* were selected and washed with M9 buffer to remove residual OP50. The supernatant was then collected by grinding, crushing, and centrifugation (3000 rpm, 10 min) in an ice bath. The levels of SOD, CAT, and GSH-px were measured following the instructions of the commercial kits. Subsequently, protein concentrations were determined using a BCA assay kit. Statistical analysis was performed using the Student’s *t*-test. The data are presented as mean ± SD.

### 2.8. RNA-seq

The L4 stage worms were cultured on NGM plates containing 100 μg/mL ARE at 20°C for 48 h. Subsequently, they were washed with M9 buffer to remove OP50, then flash-frozen in liquid nitrogen and stored at −80 °C. Afterwards, they were sent to OE Biotech (Shanghai, China) for subsequent library construction analysis. Alignment to the *C. elegans* reference genome was performed using HISAT2 software (version 2.1.0) to calculate gene expression levels (FPKM). DESeq2 software (version 1.22.2) was used to conduct differential expression gene (DEG) analysis, with DEGs defined as genes having a *p*-value <0.05 and a fold change >2 or <0.5.

### 2.9. Metabonomics

The L4 stage worms were cultured on NGM plates containing 100 μg/mL ARE at 20 °C for 48 h. Subsequently, they were washed off with M9 buffer to remove OP50, then flash-frozen in liquid nitrogen and stored at −80 °C. Afterwards, they were sent to OE Biotech (Shanghai, China) for subsequent differential metabolite analysis. Metabolites were identified based on multiple dimensions including retention time (RT), accurate mass, secondary fragmentation, and isotope distribution using The Human Metabolome Database (HMDB), Lipidmaps (v2.3), METLIN database, and the LuMet-Animal3.0 local database. Statistical significance (*p*-value) was computed using a single-factor analysis (*t*-test). Metabolites with VIP > 1 and a *p*-value < 0.05 were considered differentially expressed metabolites (DEMs).

### 2.10. Real-Time Quantitative PCR

The worms were treated with 100 μg/mL ARE for 2 days, and total RNA was extracted from the worms using the RNAiso Plus kit (Takara, Dalian, China) following the manufacturer’s instructions. Possible genomic DNA contamination in the extracted RNA was removed using the PrimeScript™ RT reagent kit with gDNA Eraser kit (Takara, Dalian, China), and then the RNA was reverse transcribed into cDNA using the same PrimeScript™ RT reagent kit with gDNA Eraser. Subsequently, real-time quantitative PCR was performed on the cDNA obtained from *C. elegans* treated with 100 μg/mL ARE, using the TB Green^®^ Premix Ex Taq™ kit (Takara, Dalian, China). The relative gene expression was calculated using the 2^−ΔΔCt^ method, with Actin-1 selected as the housekeeping gene. Primer sequences are provided in the Appendix A.

### 2.11. Nuclear Localization of DAF-16

To determine DAF-16::GFP nuclear localization, synchronized L4 larvae of DAF-16::GFP-expressing TJ356 worms were randomly divided into control and ARE groups, which cultured on NGM plates containing 100 μg/mL ARE at 20 °C for 48 h. Then, the worms (n = 20) were transferred to a 2% agarose pad on a glass slide and anesthetized by 50 μg/mL levamisole. Fluorescence microscopy was used to capture and determine the location of DAF-16::GFP (cytoplasmic, intermediate between cytoplasm and nucleus, and nuclear). Representative images and number of DAF-16::GFP nuclear localization of *C. elegans* were obtained and counted. Statistical analysis was performed using the Student’s *t*-test. The data are presented as mean ± SD.

### 2.12. Quantitation of GST-4::GFP and SOD-3::GFP Expression

The synchronized L4 stage of CF1553 and CL2166 worms were utilized to quantified the protein of SOD-3 and GST-4 expression, respectively. The CF1553 and CL2166 worms were incubated on NGM plates containing 100 μg/mL ARE at 20 °C for 2 days. Subsequently, the worms were anesthetized with 50 μg/mL levamisole hydrochloride and imaged using a fluorescence microscope (*n* = 20).

## 3. Results

### 3.1. Screening and Identification of Active Substances from A. roxburghii

**Figure 1:**
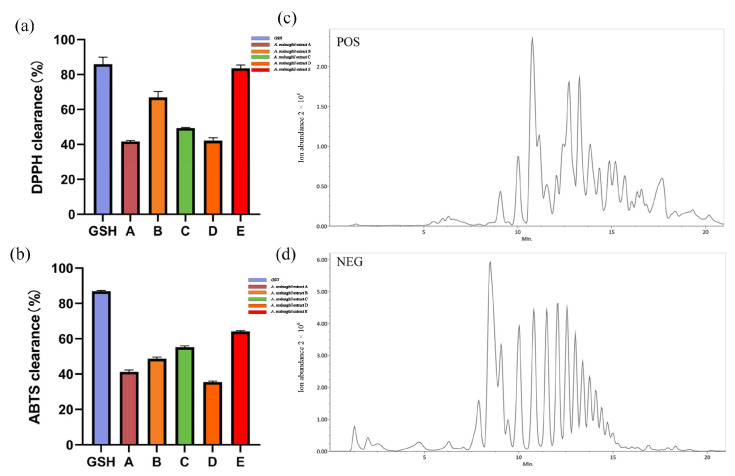
Screening and identification of active substances from *A. roxburghii*. (**a**) DPPH scavenging capacity of different 2 mg/mL extracts. (**b**) ABTS scavenging capacity of different 2 mg/mL extracts. (**c**) Positive ion chromatogram of ARE. (**d**) Negative ion chromatogram of ARE.

DPPH and ABTS assays were performed to evaluate the antioxidant capacity of *A. roxburghii* A, B, C, D, and ARE. The results demonstrated that 2 mg/mL ARE exhibited stronger scavenging abilities than 2 mg/mL *A. roxburghii* A, B, C, and D, reaching 83.55 ± 1.9% for DPPH and 64.11 ± 0.41% for ABTS (Figure 1a,b). The IC50 values of ARE for DPPH and ABTS were determined to be 0.244 mg/mL and 0.7681 mg/mL, respectively. The IC50 values for *A. roxburghii* A, B, C, and D can be found in Table A1. Therefore, ARE was selected and served as the primary active substance of *A. roxburghii*. Furthermore, UHPLC-MS/MS was employed to analyze the composition of ARE. The positive and negative total ion chromatograms of ARE are displayed in Figure 1c,d. After a detailed comparison of the mass-to-charge ratios (*m*/*z*) and secondary fragment information, a total of 65 compounds were identified in ARE (Table 1).

### 3.2. ARE Did Not Affect Food Selection, Proliferation, and Development of C. elegans

**Figure 2:**
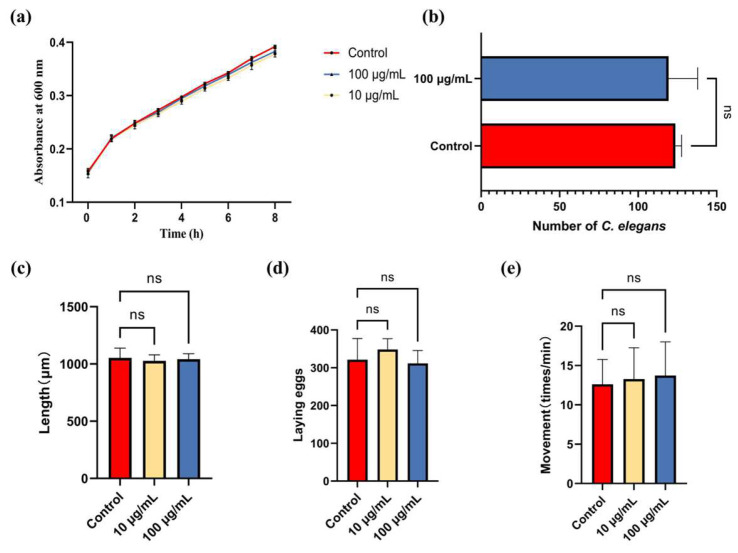
Effects of ARE on OP50 and *C. elegans*. (**a**) Effect of ARE on the proliferation of OP50. (**b**) Preference of *C. elegans* for food containing ARE (*n* = 3). (**c**) The effect of ARE on the body length of *C. elegans*. (**d**) The effects of ARE on *C. elegans* reproduction (**e**) The effects of ARE on *C. elegans* locomotion. Statistical analysis was conducted using Student’s *t*-test. Data are shown as mean ± SD. ns—not significant.

It was demonstrated that 100 μg/mL of ARE did not significantly affect OP50 proliferation (Figure 2a) and food choice of *C. elegans* (Figure 2b). Additionally, the average body length, egg laying, and movement were recorded to evaluate the impact of ARE on the growth and development of *C. elegans*. As shown in Figure 2c–e, the body length of *C. elegans* in the control, 10 of μg/mL ARE, and 100 μg/mL of ARE group was 1053.2 ± 85 μm, 1026.6 ± 52.7 μm, and 1041.3 ± 48.1 μm, respectively. The number of eggs laid by *C. elegans* in the control, 10 μg/mL of ARE, and 100 μg/mL of ARE group was 321.3 ± 56.1, 348.1 ± 28.6, and 311.7 ± 33.8, respectively. The average locomotion frequency of *C. elegans* was 12.6 ± 3.2 times, 12.6 ± 3.1 times, and 13.3 ± 4 times per 60 s in the control, 10 μg/mL of ARE, and 100 μg/mL of ARE group, respectively. These results reveal that ARE has no significant effect on the proliferation and development of *C. elegans*.

### 3.3. ARE Reduced the Accumulation of Lipofuscin and Prolonged the Life Span of C. elegans

**Figure 3:**
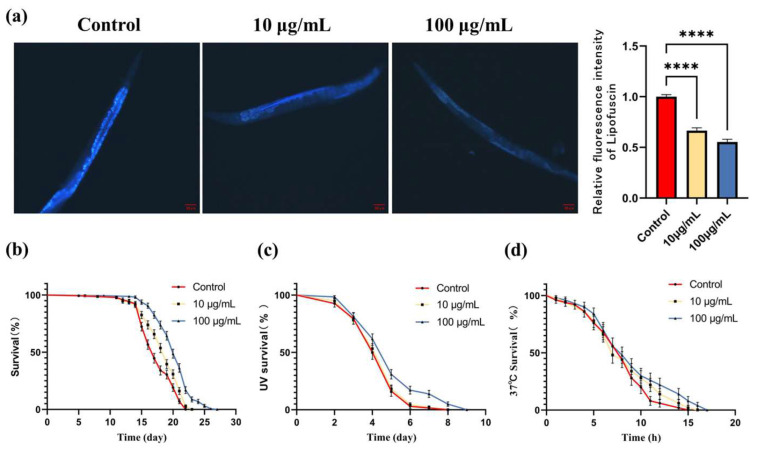
Effects of ARE on *C. elegans*. (**a**) The effect of ARE on the accumulation of lipofuscin in *C. elegans* (*n* = 20). Scale bar = 50 μm. (**b**) Effect of ARE on the lifespan of *C. elegans* (*n* = 200). (**c**) Effect of ARE on the lifespan of *C. elegans* under UV stress (n = 200). (**d**) Effect of ARE on the lifespan of *C. elegans* under 37 °C heat stress (*n* = 200). Statistical analysis was conducted using Student’s *t*-test. Data from lifespan analysis were analyzed using Kaplan–Meier analysis and a log-rank test. Data are shown as mean ± SD. **** *p* < 0.0001.

As aging progresses, lipofuscin gradually accumulates in the intestines of *C. elegans*. As shown in Figure 3a,b, compared with the control group, the relative level of lipofuscin in *C. elegans* decreased to 66.6 ± 2.7% (*p* < 0.01) and 55.3 ± 2.8% (*p* < 0.01) after treatment with 10 μg/mL and 100 μg/mL ARE. The result of the survival experiment was displayed in Figure 3c. In the control group, the median lifespan of *C. elegans* was 17 days, with an average lifespan of 17.38 days and a maximum lifespan of 22 days. In the 10 μg/mL ARE group, the median lifespan was 19 days, with an average lifespan of 18.38 days and a maximum lifespan of 23 days, resulting in a 5.7% increase in average lifespan compared to the control group. In the 100 μg/mL ARE group, the median lifespan increased to 20 days, with an average lifespan of 20.21 days and a maximum lifespan extending to 27 days, representing a 16.3% increase in average lifespan compared to the control group.

### 3.4. ARE Enhanced the Stress Resistance of C. elegans

After 0.1 J of UV irradiation, the average lifespan of *C. elegans* in the control group was 4.43 days, with a maximum survival time of 8 days. In the 10 μg/mL ARE group, the average lifespan was 4.51 days, with a maximum survival time of 8 days, showing no significant difference between the two groups. However, in the 100 μg/mL ARE group, the average lifespan increased to 5.08 days, representing a 14.7% increase (*p* < 0.01), with the maximum survival time extending to 9 days (Figure 3c).

At 37 °C, the average survival time of the control group was 7.82 h, with a maximum survival time of 15 h. In the 10 μg/mL ARE group, the average lifespan was 8.28 h, representing a 5.8% increase (*p* < 0.05), with a maximum survival time extending to 16 h. In the 100 μg/mL ARE group, the average lifespan was 9.04 h, representing a 15.6% increase (*p* < 0.01), with the maximum survival time extending to 17 h. These results indicate a significant improvement in the stress resistance of *C. elegans* treated with 100 μg/mL ARE (Figure 3d).

### 3.5. ARE Increased Antioxidant Enzyme Activity and Decreased ROS Accumulation in C. elegans

**Figure 4:**
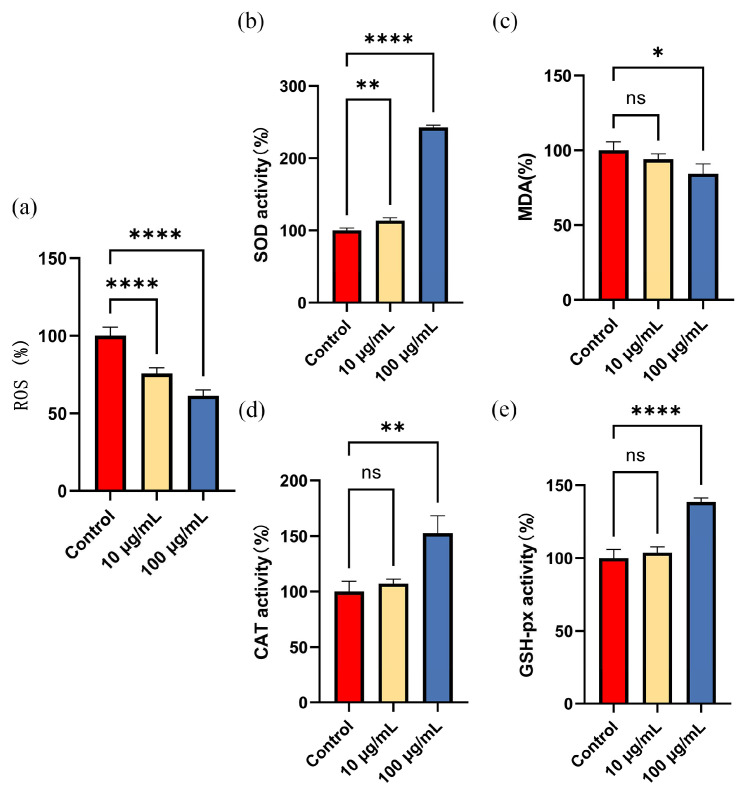
ROS content and antioxidant enzyme activity. (**a**) Effect of different concentrations of ARE on ROS accumulation in *C. elegans*. (**b**) Effect of different concentrations of ARE on SOD enzyme activity in *C. elegans*. (**c**) Effect of different concentrations of ARE on MDA content in *C. elegans*. (**d**) Effect of different concentrations of ARE on CAT enzyme activity in *C. elegans*. (**e**) Effect of different concentrations of ARE on GSH-px enzyme activity in *C. elegans*. Statistical analysis was conducted using Student’s *t*-test. Data are shown as mean ± SD. * *p* < 0.05, ** *p* < 0.01, **** *p* < 0.0001, ns—not significant.

ROS accumulation in *C. elegans* was detected after treatment with ARE for 28 h. The results demonstrated that, compared to the control group, 10 μg/mL and 100 μg/mL of ARE significantly decreased the level of ROS in *C. elegans* (*p* < 0.01) (Figure 4a).

The results of antioxidant enzyme activity showed that compared to the control group, 10 μg/mL ARE significantly increased SOD activity while having no significant effect on MDA content, CAT, and GSH-px activity (*p* > 0.05). Interestingly, 100 μg/mL ARE significantly decreased MDA and increased SOD, CAT, and GSH-px activity (*p* < 0.05). Specifically, as shown in Figure 4b–e, compared to the control group, 100 μg/mL ARE decreased MDA to 84.4 ± 6.4%, but increased SOD, CAT, and GSH-px activities to 242.7 ± 3.2%, 152.6 ± 15.7%, and 138.5 ± 2.7%, respectively (*p* < 0.01).

### 3.6. RNA-Seq and Enrichment Analysis

**Figure 5:**
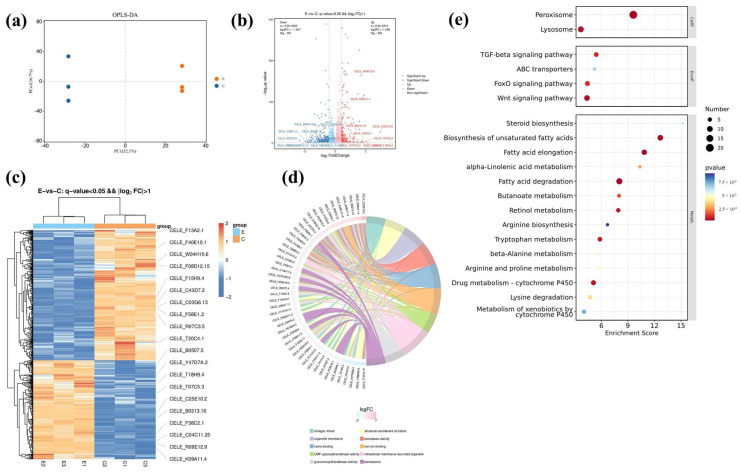
RNA seq and enrichment analysis E—100 μg/mL ARE; C—Control. (**a**) Orthogonal partial least squares discriminant analysis (OPLS-DA). (**b**) Volcano plot of significant DEGs (FC > 1.5, *p* < 0.05). (**c**) DEGs orange represents upregulation, while blue represents downregulation (FC > 1.5, *p* < 0.05). (**d**) DEGs expression GO analysis. (**e**) Differential gene expression KEGG analysis. The size of the circles corresponds to the number of DEGs and are color-coded according to −log10 (*p* value). The x-axis shows the enrichment factor value.

RNA-seq analysis was further performed to explore the potential differentially expressed genes (DEGs) and mechanisms of ARE in prolonging the lifespan of *C. elegans*. The OPLS-DA results showed that the control group and 100 μg/mL ARE group were well clustered (Figure 5a). Subsequently, compared to the control group, 581 differentially expressed genes (DEGs) were identified (*p* < 0.05, |log2FC| > 1), with 250 upregulated genes and 331 downregulated genes (Figure 5b,c). The results of GO enrichment analysis revealed that the biological process (BP) entries were predominantly linked to fatty acid beta-oxidation, xenobiotic metabolic processes, organic acid metabolic processes, and exogenous drug catabolic processes. Additionally, molecular function (MF) entries were identified and primarily associated with activities such as the structural composition of the cuticle, aromatase activity, heme binding, iron ion binding, UDP-glycosyltransferase activity, glucuronosyltransferase activity, and carboxylic ester hydrolase activity. The cellular components (CCs) entries were mainly linked to collagen trimers, organelle membranes, intracellular membrane-bounded organelles, peroxisomes, extracellular regions, and endoplasmic reticulum membranes. The BP, CC, and MF were displayed based on *p* value ranking (Figure 5d).

Furthermore, KEGG pathway enrichment analysis further highlighted the alteration of 29 related signaling pathways, significantly enriching pathways involved in peroxisome, FoxO signaling pathway, TGF-beta signaling pathway, and Wnt signaling pathway. The top 20 signaling pathways are displayed in Figure 5e. These results indicate that the expression of antioxidant enzymes in the worms was activated, and ARE might extend the lifespan of *C. elegans* through the FoxO signaling pathway.

### 3.7. Metabolomics Analysis

**Figure 6:**
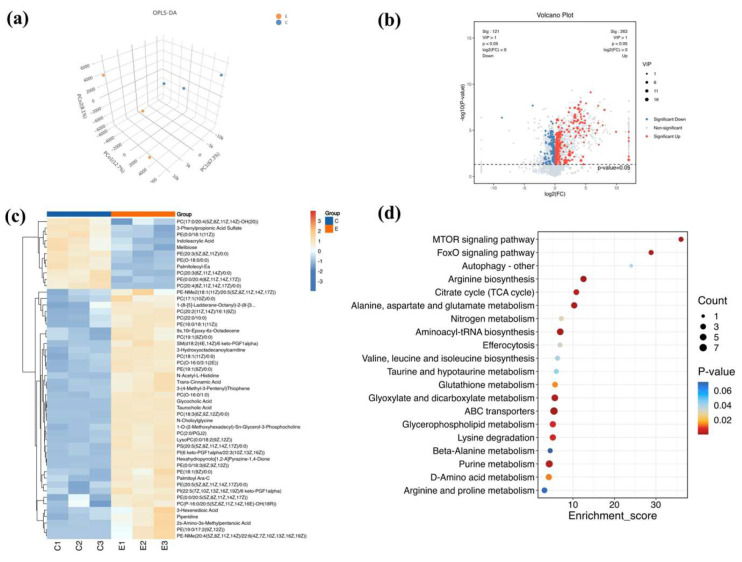
Identification of differential metabolites and enrichment analysis of metabolic pathways. E—100 μg/mL ARE. C—control. (**a**) Orthogonal partial least squares discriminant analysis (OPLS-DA). (**b**) Volcano plot of differential metabolites. (**c**) Heatmap of differential metabolites. (**d**) KEGG pathway analysis of differential metabolites. The size of the circles corresponds to the number of DEMs and are color-coded according to *p* value.

Metabolomics analysis was further performed to explore the differential metabolites and mechanism of ARE in prolonging the lifespan of *C. elegans*. The OPLS-DA results showed that the control group and 100 μg/mL ARE group were well clustered (Figure 6a). In this study, 384 differential metabolites (263 upregulated and 121 downregulated) were identified based on the criteria of *p* < 0.05 and VIP > 1.0 (Figure 6b,c). Subsequently, the results of KEGG pathway enrichment analysis showed that the top 20 pathways mainly involved ABC transporters, arginine biosynthesis, alanine, aspartate and glutamate metabolism, aminoacyl-tRNA biosynthesis, the mTOR signaling pathway, glyoxylate and dicarboxylate metabolism, and the FoxO signaling pathway (Figure 6d). Coincidentally, the FoxO signaling pathway has also been identified in metabolomics after treatment with ARE in *C. elegans*.

### 3.8. Integration of Transcriptomics and Metabolomics Networks in C. elegans

**Figure 7:**
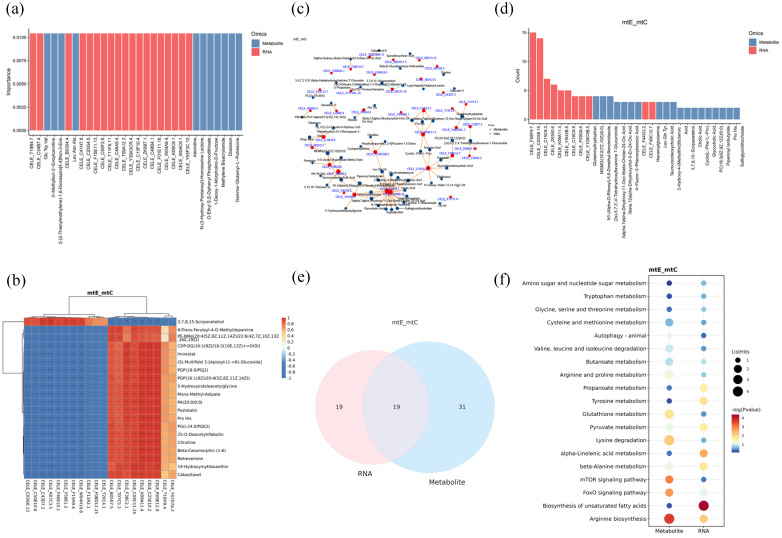
Metabonomic and transcriptome integration analysis. (**a**) Top 30 biomarkers. (**b**) Heatmap of differential genes and metabolites. (**c**) Network plot of differential genes and metabolites. (**d**) Top 30 associated network nodes. (**e**) Venn diagram of differential genes pathway and metabolites pathway. (**f**) Common differential pathways. * *p* < 0.05, ** *p* < 0.01, *** *p* < 0.001.

The random forest method was used to screen the important biomarkers. The top 30 biomarkers were displayed in Figure 7a. Furthermore, as shown in Figure 7b, the top 20 genes and the top 20 metabolites were obtained and used to build correlation heatmaps. The association network and the number of association nodes were constructed and counted by integrating the absolute value of the correlation coefficient into the top 100 genes and metabolites (Figure 7c,d). As depicted in Figure 7e, we further constructed a differential network by integrating significantly altered genes and metabolites, subsequently mapping these entities to their corresponding pathways. As a result, 19 commonly affected pathways were identified (Figure 7f), including the following: arginine biosynthesis, biosynthesis of unsaturated fatty acids, FoxO signaling pathway, and mTOR signaling pathway.

### 3.9. ARE Upregulated the Expression Levels of the daf-16, sod-3, and gst-4

**Figure 8:**
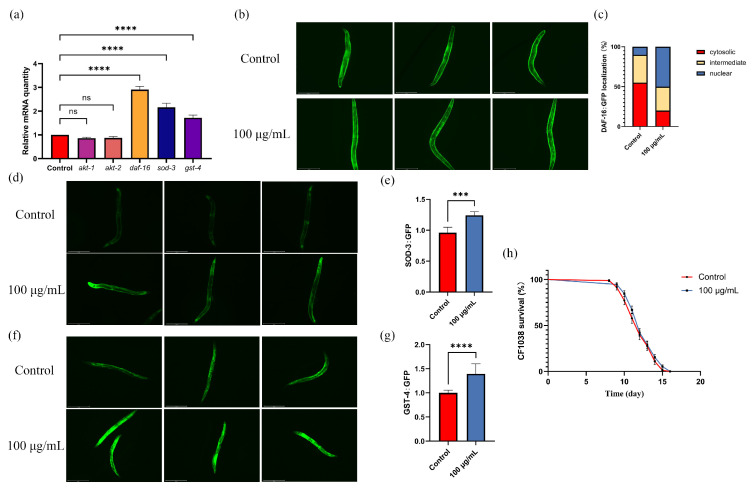
Gene expression and knockout verification. (**a**) Real-time PCR. (**b**) Fluorescent image of DAF-16. (**c**) Cellular localization of DAF-16 (*n* = 20). (**d**) Fluorescent image of SOD-3. (**e**) SOD-3 expression analysis (*n* = 20). (**f**) Fluorescent image of GST-4. (**g**) GST-4 expression analysis (*n* = 20). (**h**) Effect of ARE on lifespan of *daf-16*-deficient *C. elegans* (CF1038). *** *p* < 0.001, **** *p* < 0.0001. ns—not significant.

Real-time quantitative PCR was used to verify the expression of key genes in the FoxO signaling pathway, including *akt-1*, *akt-2*, *daf-16*, *sod-3,* and *gst-4* genes, identified through multi-omics analysis. As shown in Figure 8a, compared to the control group, ARE significantly upregulated the expression levels of *daf-16*, *sod-3,* and *gst-4* genes in *C. elegans*, while it did not significantly alter the expression levels of *akt-1* and *akt-2* genes. Specifically, the expression levels of *daf-16*, *sod-3*, and *gst-4* genes increased to 290.8 ± 13.6%, 216.2 ± 17.4%, and 139.4 ± 20.7%, respectively, after treatment with ARE compared to the control group.

Subsequently, nematode TJ356 was used to evaluate the effects of ARE on the subcellular localization of *daf-16*. As shown in Figure 8b,c, the number of nuclear-localized *daf-16* significantly increased in worms after treatment with ARE. Additionally, transgenic nematodes CF1553 and CL2166 were used to evaluate the translation of *gst-4* and *sod-3* genes downstream of *daf-16* in *C. elegans*, respectively. The results of the CL2166 and CF1553 transgenic worms showed that sod-3::GFP expression increased to 250.1 ± 50.9% (*p* < 0.01) and GST-4::GFP expression increased to 133 ± 19.7% (*p* < 0.01) (Figure 8d–g).

### 3.10. ARE Prolongs the Lifespan of C. elegans through the daf-16/FoxO Pathway

To further clarify the role of ARE in promoting the expression of downstream genes *sod-3* and *gst-4* by upregulating *daf-16*, we used *daf-16*-deficient nematodes (CF1038) to evaluate the effect of ARE on the lifespan of *C. elegans*. The results showed that the lifespan of *daf-16*-deficient nematodes treated with ARE was not significantly different from that of the control group, confirming that ARE could prolong the lifespan of nematodes by promoting the expression of *daf-16* (Figure 8h).

## 4. Discussion

In this study, we focused on *A. roxburghii*, an Asian plant with traditional medicinal and dietary uses, to explore its anti-aging properties in *C. elegans*. This research is the first to demonstrate that *A. roxburghii* can extend the lifespan of *C. elegans*. Using mutant nematode strains, transcriptomic, metabolomic, and other methods, we found that the activation of DAF-16/FoxO transcriptional nuclear activity, mediated by *A. roxburghii,* is crucial for the observed lifespan extension in *C. elegans*.

Previous studies have indicated that *A. roxburghii* possesses strong in vitro antioxidant activity [18,21], and our research has confirmed this finding. To further investigate the potential in vivo antioxidant activity of *A. roxburghii*, we used *C. elegans* as a model organism. The results showed that *A. roxburghii* extended the lifespan of *C. elegans* by 16.3%, reduced ROS and lipofuscin accumulation, and had no adverse effects on the growth, development, or reproduction of the nematodes. In summary, these results support the effectiveness of *A. roxburghii* as an in vivo antioxidant and demonstrate its bioavailability. These findings are consistent with several other studies that have clearly demonstrated the antioxidant properties of *A. roxburghii* [21,26].

To elucidate the potential mechanisms underlying the anti-aging effects on *C. elegans*, differential gene and metabolite analysis revealed significant enrichment of the FoxO signaling pathway. The FoxO signaling pathway plays a crucial role in regulating various cellular processes such as the cell cycle, proliferation, apoptosis, and antioxidant stress response [27,28]. In *C. elegans*, this pathway includes *daf-2*/IGFR, *age-1*/IP3K, and *daf-16*/FoxO [29]. Studies have shown that the increased nuclear translocation of *daf-16* is one of the main mechanisms for extending the lifespan of *C. elegans*. The nuclear translocation of *daf-16* stimulates the expression of downstream antioxidant enzymes, thereby reducing the accumulation of oxidative damage [30,31,32]. Treatment with ARE significantly increased the nuclear translocation of *daf-16* in *C. elegans* and upregulated the expression of downstream antioxidant enzymes SOD-3 and GST-4. When the *daf-16* was knocked out, the lifespan extension effect of ARE was abolished. Additionally, we found that ARE had no effect on the reproduction of *C. elegans*, confirming our hypothesis that reproductive signaling is not involved in ARE-induced lifespan extension. Therefore, we propose that ARE activates the FoxO signaling pathway, promotes the nuclear translocation of *daf-16*/FoxO transcription factors, and subsequently stimulates and regulates the expression of these two downstream proteins, SOD-3 and GST-4.

However, this study has some limitations. Firstly, it only demonstrated that ARE can activate *daf-16* to extend the lifespan of *C. elegans* and its primary and secondary metabolites have been identified. The specific relationship between these metabolites and *daf-16* remains unclear, including which substances play a role in extending lifespan and how they influence *daf-16* expression to exert their effects. Additionally, further research is needed to investigate the potential of ARE in improving age-related diseases.

In conclusion, ARE demonstrates anti-aging effects on *C. elegans* and holds potential for developing anti-aging products, providing significant benefits for middle-aged and elderly populations.

## Figures and Tables

**Table 1:** ARE composition.

Components	RT (Min)	Precursor *m*/*z*	Reference *m*/*z*	Error (ppm)	Adduct	Formula
(5E)-4-methoxy-5-[methoxy-[(2R,3S)-3-phenyloxiran-2-yl]methylidene]furan-2-one	1.158633	273.10920	273.10904	0.586	[M−H]^−^	C_15_H_14_O_5_
3-Methylxanthine	1.24045	165.04170	165.04179	0.545	[M−H]^−^	C_6_H_6_N_4_O_2_
Arabinose	1.24045	149.04580	149.04555	1.677	[M−H]^−^	C_5_H_10_O_5_
Carbetamide	1.344417	237.12330	237.12337	−0.295	[M+H]^+^	C_12_H_16_N_2_O_3_
trans-5-O-Caffeoylquinic acid	1.362117	353.10940	353.10901	1.104	[M−H]^−^	C_16_H_18_O_9_
N-Benzyloxycarbonylglycine	4.972417	208.06190	208.06154	1.730	[M−H]^−^	C_10_H_11_NO_4_
N-Isovalerylglycine	6.510334	158.08210	158.08226	−1.012	[M−H]^−^	C_7_H_13_NO_3_
3Alpha-Hydroxy-3-Deoxyangolensic acid methyl ester	6.676167	471.24510	471.24536	−0.552	[M−H]^−^	C_27_H_36_O_7_
Okaramine J_120151	6.780283	525.28610	525.28601	0.171	[M+H]^+^	C_32_H_36_N_4_O_3_
(2S,3R,4S,5S,6R)-2-[[(1S,4aR,7aS)-7-(hydroxymethyl)-1,4a,5,7a-tetrahydrocyclopenta[c]pyran-1-yl]oxy]-6-(hydroxymethyl)oxane-3,4,5-triol	6.883483	329.12400	329.12418	−0.547	[M−H]^−^	C_15_H_22_O_8_
Cytisine	6.903934	229.06770	229.06781	−0.480	[M+H]^+^	C_11_H_14_N_2_O
4-acetyloxy-8-(3-oxo-2-pent-2-enylcyclopenten-1-yl) octanoic acid	7.344133	349.11430	349.11429	0.029	[M−H]^−^	C_20_H_30_O_5_
4-[2-[(1R,4aS,5R,6R,8aS)-6-hydroxy-5-(hydroxymethyl)-5,8a-dimethyl-2-methylidene-3,4,4a,6,7,8-hexahydro-1H-naphthalen-1-yl]-1-hydroxyethyl]-2H-furan-5-one	7.344133	349.11460	349.11481	−0.602	[M−H]^−^	C_20_H_30_O_5_
MBOA	7.428117	164.03480	164.03461	1.158	[M−H]^−^	C_8_H_7_NO_3_
5,6-dihydropenicillic acid	7.428117	170.98500	170.98529	−1.696	[M−H]^−^	C_8_H_12_O_4_
1,4-Cyclohexanedicarboxylic acid	7.470117	171.06640	171.06628	0.701	[M−H]^−^	C_8_H_12_O_4_
Communesin-B	7.484583	509.29140	509.29108	0.628	[M+H]^+^	C_32_H_36_N_4_O_2_
Meglutol	7.880767	161.04580	161.04555	1.552	[M−H]^−^	C_6_H_10_O_5_
2-[5-[2-[2-[5-(2-hydroxypropyl)oxolan-2-yl]propanoyloxy]propyl]oxolan-2-yl]propanoic acid	8.028216	409.21950	409.21967	−0.415	[M+H]^+^	C_20_H_34_O_7_
Wikstromol	8.238367	307.15160	307.15158	0.065	[M+H]^+^	C_15_H_24_O_5_
Melatonin	8.245916	231.12240	231.12207	1.428	[M−H]^−^	C_13_H_16_N_2_O_2_
Isopropylmalic acid	8.733883	175.05960	175.05991	−1.771	[M−H]^−^	C_7_H_12_O_5_
Caffeine	9.06815	217.07030	217.07001	1.336	[M+H]^+^	C_8_H_10_N_4_O_2_
15,17-dihydroxy-9-methyl-4,10-dioxatricyclo [11.4.0.0,]heptadeca-1(17),13,15-triene-2,11-dione	9.096033	305.10300	305.10306	−0.197	[M−H]^−^	C_16_H_18_O_6_
(3,4,5-trihydroxy-6-methyloxan-2-yl) 2-(methylamino)benzoate	9.4975	296.11350	296.11395	−1.520	[M−H]^−^	C_14_H_19_NO_6_
MITOMYCIN C	9.4975	333.12020	333.12045	−0.750	[M−H]^−^	C_15_H_18_N_4_O_5_
Beclomethasone dipropionate	9.5138	521.23270	521.23297	−0.518	[M+H]^+^	C_28_H_37_CO_7_
Indole-3-acetyl-L-glutamic acid	9.577333	303.09860	303.09863	−0.099	[M−H]^−^	C H_16_N_2_O_5_
3′,5′-Dimethoxy-4′-hydroxyacetophenone	9.617333	195.06640	195.06628	0.615	[M−H]^−^	C_10_H_12_O_4_
3-hydroxy-2-octylpentanedioic acid	9.698167	259.15520	259.15509	0.424	[M−H]^−^	C_13_H_24_O_5_
methyl 2-benzamido-3-phenylpropanoate	9.698167	282.11370	282.11356	0.496	[M−H]^−^	C_17_H_17_NO_3_
FERULATE	9.82215	193.05020	193.05000	1.036	[M−H]^−^	C_10_H_10_O_4_
[4-[3,4,5-trihydroxy-6-(hydroxymethyl)oxan-2-yl]oxyphenyl]methyl 3-acetyloxy-2-hydroxy-2-[(4-hydroxyphenyl)methyl]butanoate	9.846617	575.15250	575.15253	−0.052	[M+H]^+^	C_26_H_32_O_12_
Shikimic acid	10.37175	197.04200	197.04204	−0.203	[M+H]^+^	C_7_H_10_O_5_
Alverine citrate	10.41242	282.22160	282.22198	−1.346	[M+H]^+^	C_26_H_35_NO_7_
[3-(4-hydroxy-3-methoxybenzoyl)-2,3-dimethyloxiran-2-yl]-(4-hydroxy-3-methoxyphenyl)methanone	10.42645	371.07480	371.07471	0.243	[M−H]^−^	C_20_H_20_O_7_
C17_Sphingosine	10.65907	286.27370	286.27399	−1.013	[M+H]^+^	C_17_H_35_NO_2_
3-[(2S,3R,4S,5S,6R)-4,5-dihydroxy-6-(hydroxymethyl)-3-[(2S,3R,4S,5R)-3,4,5-trihydroxyoxan-2-yl]oxyoxan-2-yl]oxy-5,7-dihydroxy-2-(4-hydroxyphenyl)chromen-4-one	11.07492	579.15290	579.15253	0.639	[M−H]^−^	C_26_H_28_O_15_
Styrene	11.29803	105.06960	105.06988	−2.665	[M+H]^+^	C_8_H_8_
Mollugin	11.49802	307.10000	307.10001	−0.033	[M+H]^+^	C_17_H_16_O_4_
Pesticide3_Bifenazate_C17H20N2O3_1-Methylethyl 2-(4-methoxybiphenyl-3-yl)hydrazinecarboxylate	11.53785	301.15370	301.15399	−0.963	[M+H]^+^	C_17_H_20_N_2_O_3_
8-acetamido-2-methyl-7-oxononanoic acid	11.59707	242.13950	242.13977	−1.115	[M−H]^−^	C_12_H_21_NO_4_
[(3aS,4S,5S,6E,10Z,11aR)-6-formyl-5-hydroxy-10-(hydroxymethyl)-3-methylidene-2-oxo-3a,4,5,8,9,11a-hexahydrocyclodeca[b]furan-4-yl] 2-methylprop-2-enoate	11.65785	401.09960	401.09970	−0.249	[M+H]^+^	C_19_H_22_O_7_
Licoagroside B (Not validated)	11.75722	431.11870	431.11880	−0.232	[M−H]^−^	C_18_H_24_O_12_
Scytophycin B	12.05967	842.50730	842.50702	0.332	[M+H]^+^	C_45_H_73_NO_12_
[5-hydroxy-3-(hydroxymethyl)-2-oxo-6-propan-2-ylcyclohex-3-en-1-yl] 3-methylpentanoate	12.39685	297.17090	297.17075	0.505	[M−H]^−^	C_16_H_26_O_5_
Chrysanthemic acid, ethyl ester	12.756	195.13920	195.13905	0.769	[M−H]^−^	C_12_H_20_O_2_
(2R)-2-[(2R,5S)-5-[(2S)-2-hydroxybutyl]oxolan-2-yl]propanoic acid	12.83583	215.12860	215.12888	−1.302	[M−H]^−^	C_11_H_20_O_4_
DGMG 18:3	13.1938	721.36410	721.36407	0.042	[M−H]^−^	C_33_H_56_O_14_
MEGxp0_001388	15.65933	955.29820	955.29828	−0.084	[M−H]^−^	C_41_H_32_O_27_
2-[4a-methyl-8-methylidene-4-(3-methylpentanoyloxy)-1,2,3,4,5,6,7,8a-octahydronaphthalen-2-yl]prop-2-enoic acid	15.94167	347.22250	347.22278	−0.806	[M−H]^−^	C_21_H_32_O_4_
2-(8-hydroxyoctyl)-6-methoxybenzoic acid	15.98182	279.15920	279.15900	0.716	[M−H]^−^	C_16_H_24_O_4_
Phosphatidylinositol 16	15.98182	857.51860	857.51855	0.058	[M−H]^−^	C_45_H_79_O_13_P
Carylophyllene oxide	16.02265	205.15980	205.15977	0.146	[M−H]^−^	C_14_H_22_O
Roccellic acid	16.06382	299.22230	299.22278	−1.604	[M−H]^−^	C_17_H_32_O_4_
Lauric acid	16.30847	199.17040	199.17035	0.251	[M−H]^−^	C_12_H_24_O_2_
4-({5-[5-hydroxy-3-({[(2Z)-2-methylbut-2-enoyl]oxy}methyl)pentyl]-8a-(hydroxymethyl)-5,6-dimethyl-3,4,4a,5,6,7,8,8a-octahydronaphthalen-1-yl}methoxy)-4-oxobutanoic acid	16.67412	521.31180	521.31195	−0.288	[M−H]^−^	C_29_H_46_O_8_
Ketoisovaleric acid	18.08483	117.05470	117.05462	0.683	[M+H]^+^	C_5_H_8_O_3_
Isopalmitic Acid	18.32648	257.24750	257.24750	0.000	[M+H]^+^	C_16_H_32_O_2_
FA 18:1+3O	18.38903	329.23240	329.23251	−0.334	[M−H]^−^	C_18_H_34_O_5_
Grossamide or its isomer (not validated)	18.97012	625.25680	625.25671	0.144	[M+H]^+^	C_36_H_36_N_2_O_8_
Heptadecanoic acid	19.17193	271.26040	271.26001	1.438	[M+H]^+^	C_17_H_34_O_2_
(E)-5-[(1S,4aR,8aR)-2-formyl-5,5,8a-trimethyl-1,4,4a,6,7,8-hexahydronaphthalen-1-yl]-3-(acetyloxymethyl)pent-2-enoic acid	19.32013	375.27600	375.27643	−1.146	[M−H]^−^	C_22_H_32_O_5_
6-Hydroxycaproic acid	19.77323	133.08550	133.08592	−3.156	[M+H]^+^	C_6_H_12_O_3_
Avocadene 4-acetate	20.71465	327.25420	327.25406	0.428	[M−H]^−^	C_19_H_36_O_4_

**Figure A1:**
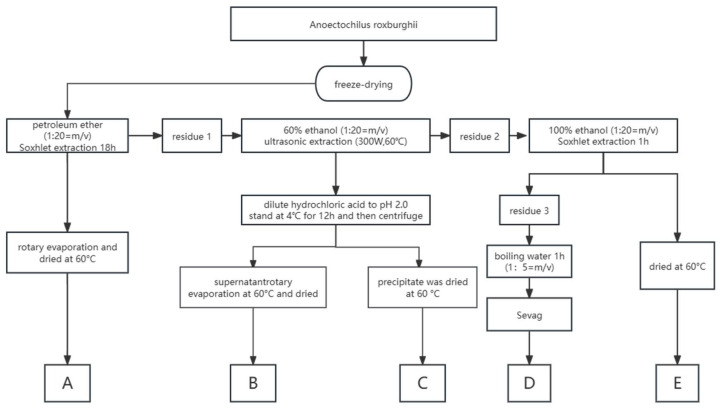
Extraction method for *A. roxburghii* flowchart.

**Figure A2:**
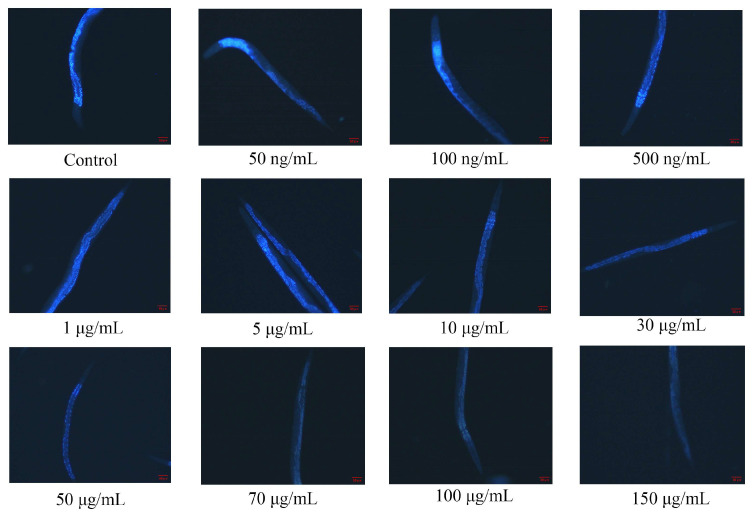
We found that the fluorescence of lipofuscin in *C. elegans* began to weaken when the concentration of ARE reached 10 μg/mL, and there was no significant change in the decrease in lipofuscin when the concentration reached more than 100 μg/mL compared with 150 μg/mL. Therefore, we chose the two concentrations of 10 μg/mL and 100 μg/mL. Scale bar = 50 μm.

## Data Availability

The raw sequence data have been submitted to the NCBI Short Read Archive (SRA) with accession number with accession number <PRJNA1132916>. The metabolomics raw data has been uploaded to the MetaboLights project number <MTBLS10402> www.ebi.ac.uk/metabolights/MTBLS10402 (accessed on 24 July 2024) [33]. The original contributions presented in the study are included in the article, further inquiries can be directed to the corresponding authors.

## Appendix A. *Anoectochilus roxburghii* Extract Extends the Lifespan of *Caenorhabditis elegans* through Activating the daf-16/FoxO Pathway

### Appendix A.1. Extraction Method for A. roxburghii

Fresh *A. roxburghii* was pre-cooled at −80 °C for 2 h, followed by freeze-drying. After freeze-drying, the *A. roxburghii* was ground into a fine powder for later use. Five grams of the dried powder were weighed and subjected to Soxhlet extraction with 100 mL of petroleum ether at a 1:20 solid–liquid ratio for 18 h, yielding residue 1 and a petroleum ether extract. The petroleum ether extract, designated as Extract A, was concentrated using rotary evaporation and dried at 60 °C. The resulting mass of Extract A after drying was 0.058 g, indicating an extraction yield of approximately 1.16%. This extract predominantly comprises non-polar or weakly polar compounds, including fats, waxes, specific steroids, and non-polar aliphatic compounds.

Residue 1 was dried and subjected to ultrasonic extraction using 100 mL of 60% ethanol at 60 °C and 300 W for 1 h. This process was repeated five times, with the filtrates combined and concentrated by rotary evaporation, yielding residue 2. The ethanol extract was divided into supernatant and precipitate fractions by adjusting the pH to 2.0 using dilute hydrochloric acid and allowing it to stand in the refrigerator for 12 h, followed by centrifugation. After concentrating the supernatant through rotary evaporation at 60 °C, it was dried to obtain Extract B, weighing 1.13 g with a yield of 22.6%. Similarly, the precipitate was dried at the same temperature to yield Extract C, weighing 0.504 g with a yield of 10.08%. Extracts B and C primarily contain highly polar compounds, such as certain organic acids, phenolic compounds, and water-soluble flavonoids.

Residue 2 was dried at a low temperature and then subjected to Soxhlet extraction using 100% ethanol at a solid–liquid ratio of 1:20 for 1 h. Subsequently, the filtered drug residue 3 was extracted with boiling water (1:5 *w*/*v*) for 1 h. It was then mixed with four volumes of anhydrous ethanol, left to precipitate at 4 °C for 12 h, and the resulting precipitate was treated with a chloroform–butanol solution in a 1:4 ratio (*v*/*v*) for three washes before drying. This product was labeled as Extract D, yielding 0.012 g with an extraction rate of 0.24%. Meanwhile, the supernatant was concentrated by rotary evaporation at 60 °C, resulting in Extract E with a mass of 0.072 g and an extraction rate of 1.44%. Extracts D and E primarily contain moderately polar compounds, such as polysaccharides, proteins, certain alkaloids, and high-molecular-weight flavonoids.

### Appendix A.2. DPPH Radical Scavenging Assay of Extracts

First, dissolve Extract A in petroleum ether. Dissolve Extracts B, C, D, and E in ethanol. Dissolve Extract F in deionized water to prepare different concentrations of the extract.

Next, prepare a 0.1 mM DPPH solution in anhydrous ethanol. Mix 3 mL of the DPPH solution with 1 mL of each sample solution, vortex thoroughly, and incubate the mixture at room temperature in the dark for 30 min to allow the free radicals in the liquid to be fully scavenged. Then, measure the absorbance of the samples at 517 nm, using a water and ethanol mixture as a blank. Use glutathione as the positive control group. The scavenging activity is calculated using the following formula:

$$Scavenging\;Rate\;(\%)=\left(1-\frac{A1-A3}{A2}\right)\times 100\%$$

A1 is the absorbance of 3 mL sample + 1 mL DPPH.
A2 is the absorbance of 3 mL sample solvent + 1 mL DPPH.
A3 is the absorbance of 3 mL sample + 1 mL ethanol.

### Appendix A.3. ABTS Radical Scavenging Assay

Prepare the ABTS stock solution by mixing equal volumes of 7 mmol/L ABTS aqueous solution and 4.9 mmol/L potassium persulfate solution. Store the mixture in the dark. Dilute the ABTS stock solution to create a series of gradient dilutions. In a microplate, add 180 μL of each diluted ABTS solution and 20 μL of deionized water. Incubate the mixture at 37 °C in the dark for 5 min, then measure the absorbance at 405 nm. Select the dilution with an absorbance value around 0.7 as the ABTS working solution for subsequent experiments (prepare fresh as needed).

Add 20 μL of different concentrations of the extract sample solution to a microplate well, followed by 180 μL of ABTS working solution. Gently vortex to mix and incubate at 37 °C in the dark for 5 min. Measure the absorbance at 405 nm (A1). Use the sample solvent instead of the extract solution for the blank control (A2), and use distilled water instead of the ABTS working solution for the zero-adjustment control (A3). Use reduced GSH at the same concentration as the positive control group. The scavenging activity is calculated using the following formula:

$$Scavenging\;Rate\;(\%)=\left(1-\frac{A1-A3}{A2}\right)\times 100\%$$

**Table A1:** Different Anoectochilus roxburghii extracts IC50.

*Anoectochilus roxburghii* Extract	DPPH (IC50)	ABTS (IC50)
A	0.2068 mg/mL	4.872 mg/mL
B	0.2692 mg/mL	5.942 mg/mL
C	0.0488 mg/mL	2.107 mg/mL
D	0.1471 mg/mL	9.961 mg/mL
E	0.0319 mg/mL	0.8797 mg/mL
